# Towards Raman imaging of centimeter scale tissue areas for real-time opto-molecular visualization of tissue boundaries for clinical applications

**DOI:** 10.1038/s41377-022-00828-2

**Published:** 2022-05-19

**Authors:** Oleksii Ilchenko, Yurii Pilhun, Andrii Kutsyk

**Affiliations:** 1 Technical University of Denmark, Department of Health Technology, Center for Intelligent Drug Delivery and Sensing Using Microcontainers and Nanomechanics, Kgs, Lyngby, 2800 Denmark; 2 Lightnovo ApS, Birkerød, 3460 Denmark; 3 Taras Shevchenko National University of Kyiv, Department of Quantum Radio Physics, Kyiv, Ukraine; 4 Technical University of Denmark, Department of Energy Conversion and Storage, Kgs, Lyngby, 2800 Denmark

**Keywords:** Raman spectroscopy, Imaging and sensing

## Abstract

Raman spectroscopy combined with augmented reality and mixed reality to reconstruct molecular information of tissue surface.

## Graphical abstract

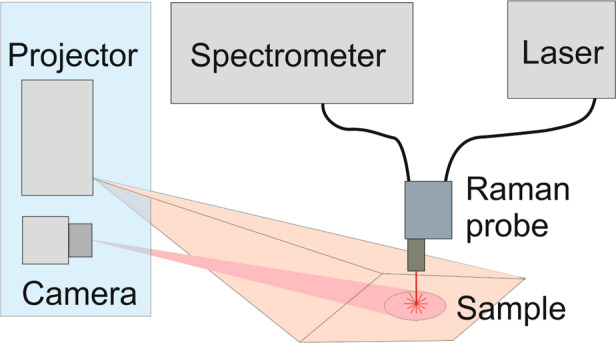

There is a growing interest in the development of methods for real-time diagnostics of diseases inside and outside of the human body. For example, screening of patients for cancer and localization of suspicious lesions for surgical excision can be performed by magnetic resonance imaging, computed tomography, positron-emission-tomography, and ultrasound. However, these methods provide information based on morphological or anatomic differences of the tissue, disregarding the underlying molecular composition. This is where the potential of Raman spectroscopy as a non-invasive, label-free, non-contact technology can be used. Raman spectroscopy is based on an inelastic scattering event between a photon and a molecule, providing an intrinsic molecular fingerprint of a sample^1^. Nowadays, the Raman instrumentation required for high-quality Raman measurements of tissue is still relatively bulky to allow direct access to the patient site. Unfortunately, commercially available handheld Raman spectrometers do not provide sufficient signal quality of the Raman spectra of tissue due to low Raman cross section and laser/Raman beam attenuation in scattering tissue media^2^. Therefore, fiber optic probe-based Raman systems are used since these allow direct access to the patient site^3^. This technology has been proven to be able to perform tissue diagnostics, detecting and differentiating cancer from healthy tissues^4–6^. However, fiber-guided Raman spectroscopy is limited to single-point measurements, while many applications require spectroscopic imaging of the area on the sample. To solve this problem multiple solutions have been developed; they include multiple optical fibers^7^, passively coordinated mechanical arms^8^, robotic arms^9^, and computer vision-based positional tracking approaches^10^. The discussed approaches provide raw Raman spectra and therefore do not facilitate additional necessary disease diagnostics information without postprocessing. A recent publication by Yang et al.^11^ has combined development of image-guided fiber optic probe-based Raman spectroscopy and real-time data-processing algorithms. The authors developed a combined Raman acquisition process with augmented reality (AR) and mixed reality (MR) to enhance the perception of molecular information. This could enable the visualization of the molecular distribution in clinical applications. The proposed and experimentally demonstrated fiber-optic probe-based imaging system enables a non-destructive and label-free acquisition of molecular images from a large tissue sample. The authors also implemented a data-processing engine into the acquisition flow, enabling an evaluation of the Raman signatures of complex biochemical macromolecules in real-time and a visualization of molecular virtual reality, i.e., augmented reality and mixed reality. Additionally, for the application to 3D surfaces, an assessment of topography through photometric stereo technique was implemented, allowing a mapping of the reconstructed molecular information on a 3D model of the sample (see method illustration in Fig. 1). This way authors combined several methods which they were developing for a few years to bring Raman spectroscopy closer to clinical applications^10^. Their system automatically tracks position of the laser spot from Raman probe during measurements, data is processed in real-time, and identification results showing chemical composition are projected as visible image directly on the sample creating mixed reality picture. It is considerable improvement over conventional approach, when the sample is analyzed at separate laboratory facility and results are available only later after mapping and full data processing is done.

**Fig. 1:**
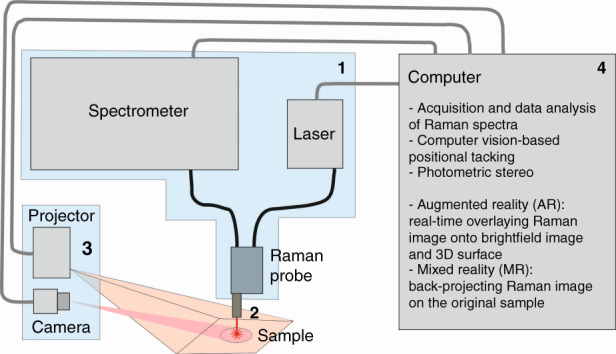
Schematic of a fiber-guided Raman system combined with augmented reality (AR) and mixed reality(MR) for real-time opto-molecular visualization of tissue boundaries for clinical applications.

The developed methodology^11^ was applied on the visualization of molecular boundaries of pharmaceutical compounds in porcine brain, brain-tumor phantom, and various types of sarcoma. The method will very likely be used in many other clinical applications as for example wound bacteria identification by Raman spectroscopy^12^. Future method development could be associated with both hardware and data-processing innovations. Perspective innovations may involve four main directions.

## Miniaturization and cost reduction

Further development is anticipated to be related to miniaturization of the Raman spectrometer including laser/Raman beam delivery optics (part 1 in Fig. 1) down to the size of a Raman fiber probe. In this way, fiber optics would not be needed to allow direct access to the patient site. The absence of fibers will also increase Raman signal throughput from sample to detector. Moreover, a compact Raman system will significantly minimize the cost of in-vivo diagnostics by Raman spectroscopy. Possible miniaturization strategies have already been proposed^13^. Other authors were able to minimize the size of a Raman spectrometer down to several centimeters while maintaining a high sensitivity of the system^14^.

## Optics optimization

Optimization of laser excitation wavelength depending on the application may significantly improve the quality of the signal. The commonly used excitation wavelength for Raman spectroscopy of tissue is 785 nm. However, lasers with longer wavelengths may be applied, which will minimize unwanted fluorescence background. This may be critical for skin cancer diagnostics where Raman signals need to be collected from dark tissue areas^15,16^.

The development of immersion probes with a high numerical aperture that provides a small laser spot size (part 2 in Fig. 1) will generate optimized conditions for laser/Raman beam propagation in/out of tissue and provide a matching of the reflection index between the last optical surface of the probe and tissue media. The small laser spot size will increase the signal-to-noise ratio of Raman and decrease the fluorescence background due to different responses of Raman and fluorescence signal in regard to the increased laser power density^17^. Nevertheless, immersion probe will require the development of disposable tips to avoid contamination of tissue.

## Visualization and data merging

We expect more advanced ways of obtaining visible image along with Raman map of the sample to appear. The Raman probe can be combined with the optical sensor in a single unit, providing more precise probe tracking and control of sample-to-probe distance that may include autofocusing^18^.

Integration of fiber-guided Raman probe into the endoscopic probe for in-body molecular analysis supported by microscopy images seems advantageous. Combination with other optical modalities such as optical coherence tomography (OCT), hyperspectral imaging, second harmonic generation, and Coherent Anti-Stokes Raman Scattering (CARS) microscopy will also be beneficial for enhanced diagnostics.

## Data analysis

The complexity of biological samples causes the complexity of Raman spectra which require chemometrics or machine learning data analysis for features extraction^19,20^. Raman spectroscopy combined with modern data analytics provides a fast and reliable tool for non-invasive biomedical diagnostics^21^.

To obtain quantitative information about investigated sample non-negative least squares (NNLS) can be used when spectra of possible components are known as it was done in the recent publication^11^ (part 4 in Fig. 1). Otherwise, different non-negative matrix factorization (NMF) techniques could be used^22^. For example, multivariate curve resolution using alternating least squares (MCR-ALS) is used widely due to its efficiency and simplicity in incorporating prior information about an investigated sample^23^. Combining with confocal Raman microscopy NMF techniques provide a spatial mapping of chemical components over samples^17,24–27^.

Significant increases in data sizes lead to difficulty in calculation and may prevent subtle features extraction in a big dataset. It makes the application of chemometric techniques challenging. Deep learning seems to be a perspective tool for big data analysis^28–30^. It can be used not only for typical classification and regression problems but for data preprocessing, improving image resolution^31^, and providing predictive models for biochemical data^29^.
